# Gastrointestinal Parasites of Zoonotic Importance Detected in Bats in the Conservation Area of Semuliki National Park, Western Uganda

**DOI:** 10.1155/japr/9972163

**Published:** 2025-08-15

**Authors:** James Robert Ochieng, Charles Drago Kato, John Joseph M. Kisakye

**Affiliations:** ^1^Department of Zoology, Entomology and Fisheries Sciences, College of Natural Sciences, Makerere University, Kampala, Uganda; ^2^College of Veterinary Medicine, Animal Resources and Biosecurity, Makerere University, Kampala, Uganda

**Keywords:** bats, gastrointestinal parasites, Semuliki National Park, Uganda, zoonosis

## Abstract

Bat guano may contain zoonotic parasites that contaminate the environment and/or serve as a potential source of infection to humans and animals. Repeated bat–human exposure could be a risk factor for zoonosis. To date, knowledge on the status of bat gastrointestinal parasites (GIPs) in Uganda is limited. We conducted a cross-sectional study to investigate the prevalence of bat GIP species in communities contiguous to Semuliki National Park (SNP), Bundibugyo district in western Uganda. We purposively collected faecal samples of micro- (*n* = 242) and megabats (*n* = 242) from bat roosts in communities contiguous to SNP during the rainy months of October to December 2023 and the dry months of January to March 2024. Standard faecal floatation and sedimentation techniques were used for laboratory examination. Microscopic examination revealed that 224 (46%) samples tested positive for more than one parasite species. Thirteen GIPs, including protozoa (*n* = 3), trematode (*n* = 1), cestode (*n* = 1) and nematodes (*n* = 8), were detected. The most prevalent parasites were *Entamoeba coli* (57%), hookworm (33%), *Strongyloides* sp. (33%) and *Entamoeba histolytica* (32%), and the least prevalent were the two unidentified nematodes (1%). Seventy-seven percent (*n* = 10) of the detected GIPs are responsible for zoonosis and are of significant public health importance. Statistically, there was a significant difference (*p* < 0.05) in the overall parasite prevalence across the four studied bat groups. Also, parasite prevalence was significantly higher in microbats compared to megabats (*p* < 0.05) and in Burondo subcounty compared to Ntandi town council (*p* < 0.05), though seasonality did not have a significant impact. The detected zoonotic parasites pose a potential source of zoonosis in communities contiguous to the conservation area of SNP, Uganda. This calls for awareness creation on the risks of bat-mediated zoonotic parasitosis and the use of good sanitary practices to prevent chances of zoonotic parasite spillover from bats to humans.

## 1. Background

Gastrointestinal parasitic infections in wildlife can be influenced by abiotic and biotic factors and parasites' ecology [1, 2]. GIPs play a major role in ecosystems, affecting hosts' ecology, the evolution of interspecific interactions, population growth and fitness and increasing vulnerability to diseases and/or fatality if not treated [3–6]. Globally, GIPs have been recognized as causing significant morbidity and mortality in wild fauna, including bats [3, 7], and are therefore essential parasites to consider in wildlife conservation [5]. The current climate change, along with other drivers like an increase in human population, changes in land use, road construction projects, forest penetration and destruction of parasite reservoirs' natural habitats, natural calamities and illegal hunting, threatens bat populations [7]. These factors increase the risks of human–bat interaction, yet bats play a significant role in pathogen transmission [6, 8].

Bats (order Chiroptera) are the only active flying true placental mammals of the animal kingdom and are the second largest order of mammals after rodents (order Rodentia) with cosmopolitan distribution [9, 10]. Traditionally, bats are classified into two major groups: suborder Megachiroptera (megabats), the fruit-eating bats (fruit bats), and suborder Microchiroptera (microbats), the insectivorous bats [11, 12].

The megabats are much larger herbivores, and they consume plant fruits, flowers, leaves, nectar and pollens and are commonly seen in fruiting trees where they roost in tightly packed clusters [9–12]. In contrast, the microbats are mostly insectivorous, though a few of these species may also feed on blood, fruits, nectar, pollens and vertebrates [9, 10]. Microbats are more common and numerous than megabats and usually hang in caves, on roofs and in tree hollows during the day [10].

Globally, more than 1400 bat species have been reported [8, 12], although many are currently threatened, and over 289 species are categorized as endangered, vulnerable or near threatened by the International Union for Conservation of Nature (IUCN) Red List [8]. Studies by [12] documented 90 bat species: 13 megabats and 77 microbats in Uganda, with over 10 species suspected to be in the conservation areas of Semuliki in Bundibugyo district, western Uganda. In Uganda, bats are widespread throughout the country and are particularly abundant in the conservation areas, suburban and urban areas, including in domestic settings, human buildings, hospitals, churches and school premises [13]. Other bat roosts are known to inhabit different environmental settings, including caves, disused mines, rock crevices, tree hollows and holes and termite nests [13, 14]. However, the bats' ecology varies and is peculiar with their feeding behaviour, which also determines the risks of bat–parasite and bat–human interactions [8].

In the aspect of public health, over 70% of new, emerging and re-emerging infectious diseases are of animal origin, and research has shown that bats play a central role in the ecosystem by serving as carriers, reservoirs, and/or transmitters of pathogens of public health importance globally [4]. The bat-transmitted pathogens include fungi, bacteria, viruses and parasites, which can affect humans and/or animals [6, 8, 15]. Bats may transfer these pathogens over long distances as they move from sylvatic to domestic settings and vice versa whilst seeking food and other basics and as they share shelter with humans and other animals [6, 8].

Currently, Uganda has documented a total of several bat-mediated pathogens, including numerous rabies, Marburg viral haemorrhagic fever [16] and eight Ebola outbreaks involving the districts of Gulu (2000), Bundibugyo (2007), Luwero (2011 & 2012), Kibaale (2012), Luwero (2012), Mubende and Kasanda (2022) and Kampala (2025) since 2000 [13, 17, 18]. The recent Marburg outbreak in the Kween district, eastern Uganda, was traced to rock salt mining in a bat cave [13], and the Egyptian fruit bat, *Rousettus aegyptiacus*, was identified as a Marburg virus reservoir [19]. However, these zoonotic bat-associated pathogenic viruses still need more attention for human and animal health in Uganda and beyond and are also critical for wildlife conservation.

Bat parasitosis studies in East Africa, including Kenya [10], other African countries [20, 21] and beyond Africa, documented several GIPs, including those responsible for zoonosis. Studies by [22] reported 59 species of helminths: 28 nematodes, 23 trematodes, 6 cestodes and 2 acanthocephalans in Brazil. Adhikari et al. [10] reported *Ascaris* spp., *Capillaria* sp., *Cryptosporidium* sp., *Eimeria* spp., *Entamoeba* sp., *Giardia* sp., *Hymenolepis* spp., *Isospora* sp., *Oxyurid* sp., *Strongyle* and *Strongyloides* sp. in insectivorous bats and *Eimeria* sp., *Entamoeba* sp. and *Hymenolepis* sp. in frugivorous bats in Kenya. Okafor et al. [21] detected five nematodes, two trematodes and two cestodes in microbats in southeast Nigeria. Saoud and Ramadan [20] described several nematodes, trematodes and cestodes in micro- and megabats in Egypt. Kváč et al. [23] found *Cryptosporidium* spp. in bats from the United States and the Czech Republic. The authors of [24, 25] observed *Hymenolepis* spp. in bats in China and Japan, respectively. McAllister and Upton [26] discovered two *Eimeria* spp. in microbats of the family Vespertilionidae: eastern red bat (*Lasiurus borealis*) in North Carolina, whilst [27] recognized six *Eimeria* spp. in Vespertilionid bats in North America. The variations in bat GIP diversity and/or prevalence may be due to differences in the status of the sampled bats, geographical location, seasonal variations and the diagnostic techniques used [6, 8]. Other related studies documented bat ectoparasites, including ticks, fleas and bed bugs [8, 28–30].

Bat parasitic infections may range from minimal to advanced effects and can significantly affect their fitness, depressing their metabolism, as has been reported elsewhere [7, 8]. These parasitic infections suppress bats' physiological and immunological responses, leading to reduced movement/flight capability, breeding success and increased inactivity, increasing their vulnerability to predation and other diseases and resulting in death in most cases [6–8, 31]. This is critical for wildlife conservation and can lead to biodiversity loss in addition to public health risks.

Currently, several bat roosts consisting of over 300 bats have been reported in human settings: homes, schools, hospitals, churches, trees (like cocoa, mangoes and avocadoes), disused mines, rock crevices and termite nests in Burondo subcounty and Ntandi town council Bundibugyo district, Uganda, by the Uganda Wildlife Authority (UWA), STOP Spillover team [13] and community members. This may worsen due to climate change, environmental shifts, amongst other factors. The bats pose a nuisance and health risks to humans. Surprisingly, up-to-date, little is known about the bat GIPs inhabiting the conservation area of SNP. Understanding the status of bat GIPs in communities contiguous to SNP is crucial for implementing effective control and prevention strategies for potentially zoonotic parasite spillover from bats to humans. This study is aimed at broadening knowledge on the current status of bat GIPs in bat hotspots in human communities contiguous to SNP to prevent the chances of potentially zoonotic parasite spillover from bats to humans. In this study, the potentially zoonotic parasites of public health importance are GIPs that can be transmitted from animals, including bats, to humans and cause illness like diarrhoea, malabsorption, anaemia and growth retardation in young children. Severe infections may lead to intestinal obstruction, perforation, amongst other serious complications. The study objectives were to quantify the diversity and prevalence of bat GIPs and to assess the risks of bat-mediated zoonotic parasitosis. Given the present limitations to national surveillance of wildlife-mediated parasitoses in Uganda, including scarce resources, focusing on forecasting spillover dynamics of bat-mediated zoonoses using a simple approach, ‘bat guano diagnosis' could be a wise alternative. This study will contribute more knowledge on the bat GIP species diversity and the risks of zoonotic parasitoses in communities contiguous to SNP Bundibugyo, Uganda. This will increase awareness of the risks of bat–human interaction in human communities to save lives and strengthen biodiversity conservation.

## 2. Materials and Methods

### 2.1. Study Area

**Figure 1:**
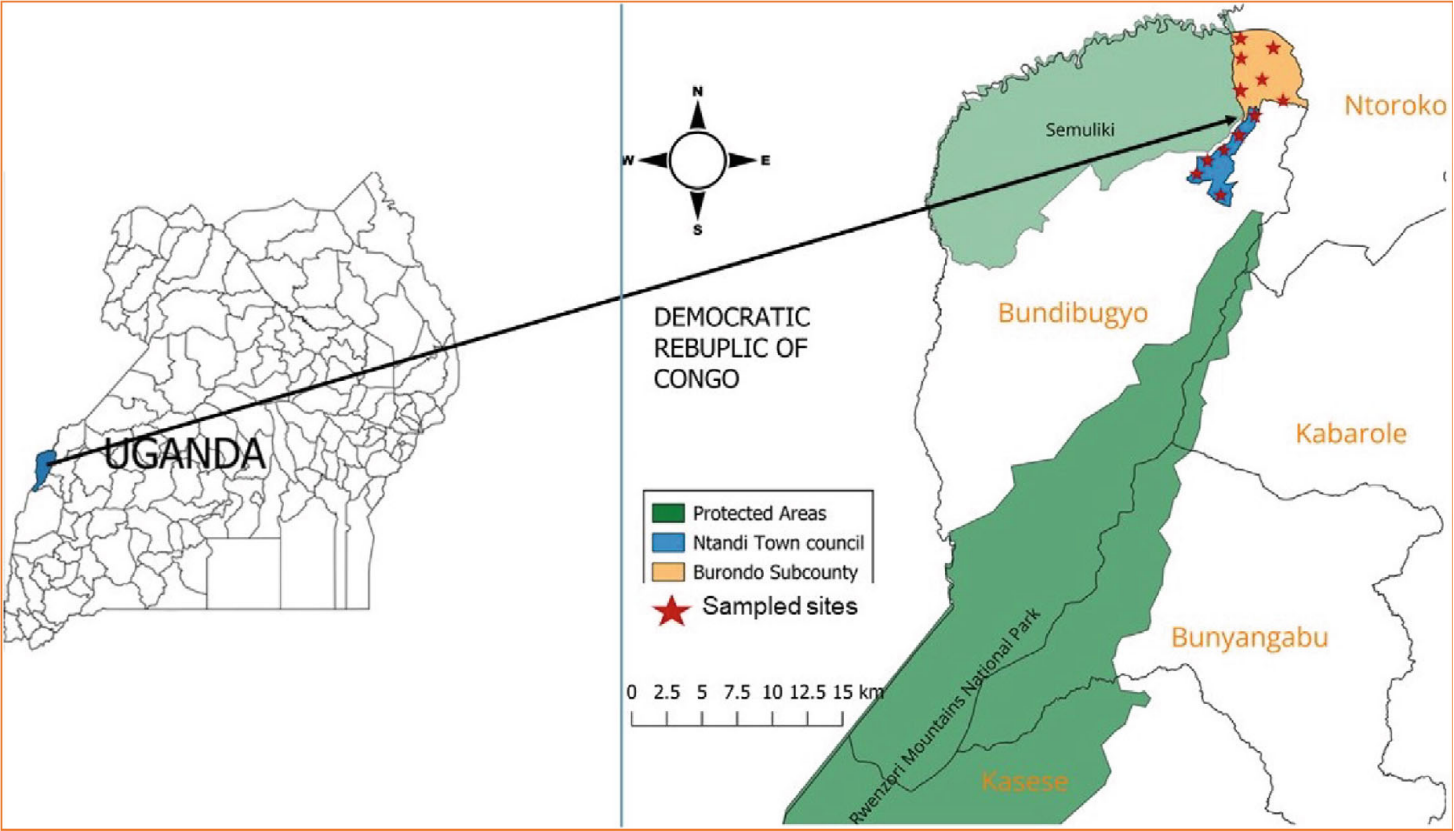
Map of the study area showing the location of Ntandi town council and Burondo subcounty in Bundibugyo district, western Uganda. The red stars show the sampled sites within the study area.

The study was carried out in human communities in Burondo subcounty and Ntandi town council contiguous to SNP in Bundibugyo district, western Uganda (Figure 1). The study area, being contiguous to the park, possesses a high level of human–wildlife–forest ecosystem interactions. Bundibugyo landscapes have plenty of rock shelters and caves that are habitats for wildlife, including bats [13]. Bundibugyo receives an average rainfall of 1250 mm, with rainfall peaks from March to May and from September to December, characterized by excessive flooding.

SNP covers an area of 220 km^2^ and lies between 0″ 44⁣′–0° 53⁣′ N and 29″ 57⁣′–30° 11⁣′ E with an altitudinal range of 670–760 m above sea level and a 18°C to 30°C (64°F to 86°F) temperature range, with relatively small daily variations. Semuliki Forest was made a National Park in October 1993, making it one of Uganda's newest National Parks, and is the only remaining primary lowland (ranging from 670 to 760 m above sea level) tropical rainforest in East Africa [29]. SNP is contiguous with undisturbed forests of the Congo Basin, including Virunga National Park in the Democratic Republic of Congo to the west, and is known to be rich in biodiversity, possessing many butterfly species and more than 441 bird species, with 31 species including capuchin babbler, piping hornbill, blue-headed crested flycatcher, red-bellied malimbe and orange weaver, known to occur nowhere else in East Africa [30] and over 10 bat species. Due to the current increase in the human population in Uganda, SNP, amongst other protected areas, remains under threat by agriculturalists and wild hunters/poachers, despite the conservation efforts by the UWA and stakeholders. Preliminary investigation by the UWA, STOP Spillover team [13] and communities has identified Burondo subcounty and Ntandi town council as hotspot places with a significant population of bats. Furthermore, bat poachers have been reported by UWA in the communities of Burondo subcounty and Ntandi town council in contiguity to SNP. The bat poachers go to the park to collect firewood, poles, palm oil and grass for domestic use.

### 2.2. Study Design and Field Faecal Sample Collection

We applied a cross-sectional study design. Batches of bat faecal samples were purposively collected from bat roosts in communities in Burondo subcounty and Ntandi town council in contiguity to SNP Bundibugyo district, western Uganda. Samples were collected during the wet months of October to December 2023 and the dry months of January to March 2024. The sample size was determined using Thrusfield's standard formula [32]. Data collection involved both bat faecal sample collection and the use of a questionnaire for risk factors.

**Figure 2:**
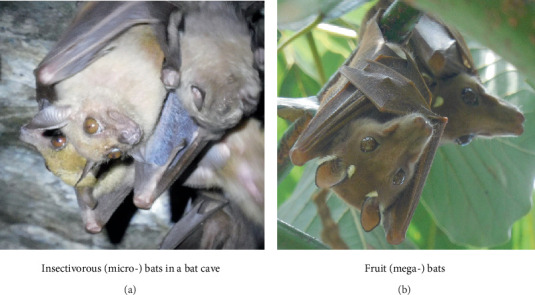
Photograph of (a) a colony of micro- (insectivorous) bats in a roost resting in a rocky bat cave and (b) two megabats (Egyptian fruit bats, *Rousettus aegyptiacus*) resting on an avocado tree in Burondo subcounty, Bundibugyo. *Original photos by James Robert Ochieng.*

Briefly, during the bat faecal sample collection, at least a total of 40 clean white polyester sheets of cloth were overlaid on the floor of five bat roosts: human houses (*n* = 2), fruit (cocoa, mangoes and/or avocadoes) trees (*n* = 2) and rock crevices/caves (*n* = 1) in Burondo subcounty and Ntandi town council every morning at 6:00 AM East African time. With the help of forceps, bat faecal samples (≥ 0.5 g each) that fell on the white polyester sheets were collected daily at midday and evening 6:00 PM and placed in clean 10-mL sterile vials, where they were thoroughly mixed with 10% formalin for preservation at room temperature until laboratory analysis at the Zoology Department, Makerere University, Kampala, Uganda. Sample collection was repeated during the wet and dry seasons until a sample size, *n* = 121, for micro- and megabats (Figure 2) was obtained per season, totalling 484 samples for both seasons. Quality control, such as observing the presence of other mammals inside the bat roosts, was taken into consideration. Sociodemographic data and detailed information on the season, GPS coordinates, roost abundance, human economic activities, lifestyle hygiene and sanitation in the study sites were all captured.

### 2.3. Parasite Identification

All the collected bat faecal samples were analyzed; each sample was divided into two portions and subjected to floatation with sodium nitrate (NaNO_3_) and sedimentation with formol-ether standard methods [33] before microscopic observations. Briefly, 4 mL of the saturated NaNO_3_ solution was added to 1 mL of 10% formalized faecal sample in a beaker and stirred with an applicator before being filtered through a gauze mesh into a second beaker. The filtrate was poured into a 2-mL vial and filled to the brim with NaNO_3_, forming a convex meniscus. A cover slip was gently placed on top of the meniscus, avoiding any air bubbles, and left undisturbed for at least 5 min, enabling the parasite eggs, cysts and/or oocysts to float to the surface whilst the heavy faecal debris sank. The cover slip was then carefully lifted upwards by a straight pull and gently placed on its face downwards on a labelled glass slide stained with Lugol's iodine. The slide was then examined under the microscope at 10× and 40× objective lenses for parasite identification. The GIP eggs, cysts and trophozoites were identified using approved structural and morphological principles based on the size, colour, shape, contents, thickness of the shell and the presence or absence of specialized structures such as knobs, opercula and/or spines [15, 28]. A calibrated ocular micrometre was used to measure the length and width of individual cysts, eggs and/or oocysts.

Sedimentation involved adding 2 mL of formalized faecal sample to 5 mL of formol-ether, mixing and filtering through a 350 *μ*m gauze mesh. The filtrate was poured into a 15-mL centrifuge tube, followed by ≥ 3 mL of diethyl ether to make 10 mL, sealed and vortexed. As prescribed by [33], the mixture was then centrifuged at 1500 rpm for 5 min to form four layers: the supernatant (top three layers), which was discarded, leaving the sediment layer suspected to contain heavy and/or operculated eggs. About 10 *μ*L of the sediment was pipetted onto a glass slide, and a drop of Lugol's iodine stain was added, covered with a coverslip and examined under the microscope at 10× and 40× objective lenses to detect parasites as was the case during floatation.

### 2.4. Statistical Analysis

All the statistical tests were performed using the IBM SPSS Statistics 23 package. Whilst GIP species diversity was defined as the total number of parasite species detected during the study, GIP prevalence was defined as the proportion of parasite-infected bats to the total number examined (Table 1). Species richness meant the number of different GIPs in bats per site, and evenness meant the relative abundance of GIPs detected in bats per site; these were calculated using the Shannon–Wiener diversity index (Table 2). The Pearson chi-square test was used to assess any statistical difference in the overall GIP prevalence rate between micro- and megabats in Burondo subcounty and Ntandi town council. The Mann–Whitney *U* test was used to assess any statistical difference in the overall GIP prevalence rate between micro- and megabats and wet and dry seasons during the study. Confidence intervals (95%) and *p* < 0.05 were set for significance.

## 3. Results

**Figure 3:**
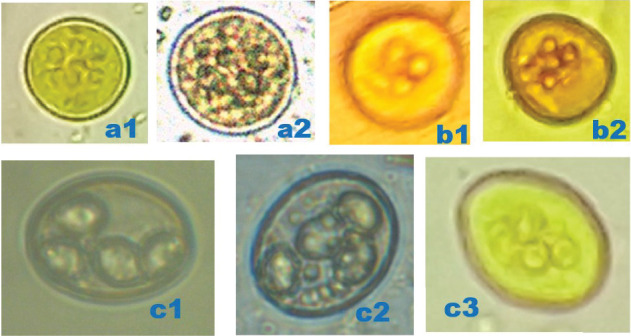
Photomicrographs of protozoan parasites detected in bats. (a1) *Entamoeba coli* cyst (10 × 10 *μ*m), 400×, after floatation technique, in microbat. (a2) *E*. *coli* cyst (12 × 12 *μ*m), 400×, after floatation technique, in megabat. (b1) *E*. *histolytica* cyst (11 × 11 *μ*m) with four nuclei, 400×, after floatation technique, in microbat. (b2) *E*. *histolytica* cyst (10 × 10 *μ*m) with four nuclei, 400×, after floatation technique, in megabat. (c1) *Eimeria* sp. oocyst (14 × 14 *μ*m) with four sporocysts, 400×, after floatation technique, in microbat. (c2) *Eimeria* sp. oocyst (14 × 14 *μ*m) with four sporocysts, 400×, after floatation technique, in megabat. (c3) *Eimeria* sp. oocyst (14 × 14 *μ*m) with four sporocysts, 400×, after sedimentation technique, in microbat. *Original micrographs by James Robert Ochieng.*

**Figure 4:**
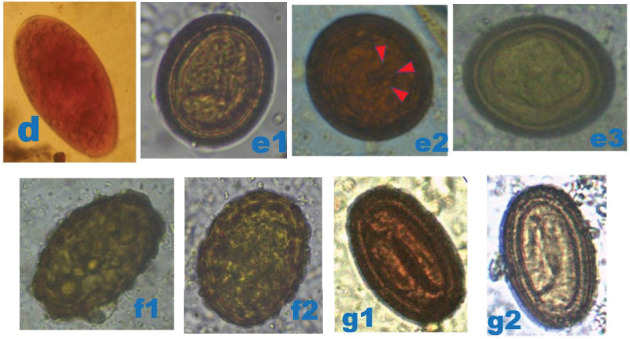
Photomicrographs of helminthic parasites detected in bats. (d) Ellipsoidal *Fasciola* sp. egg (95 × 50 *μ*m), 400×, after sedimentation technique, in microbat. (e1, e2) Large round *Hymenolepis* sp. egg (52 × 52 *μ*m), 400×, after floatation technique, in microbat; the red arrowheads show the three small paired hooks. (e3) Oval *Hymenolepis* sp. egg (45 × 35 *μ*m), 400×, after floatation technique, in microbat. (f1) Unfertilized *Ascaris* sp. egg (75 × 40 *μ*m), 400×, after floatation technique, in microbat. (f2) Fertilized *Ascaris* sp. egg (65 × 60 *μ*m) with a thick shell having an external mamillated layer/rippled surface, 400×, after floatation technique, in megabat. (g1, g2) *Chitwoodspirura* sp. eggs (70 × 45 *μ*m), 400×, after floatation technique, in microbat and megabat, respectively. *Original micrographs by James Robert Ochieng.*

**Figure 5:**
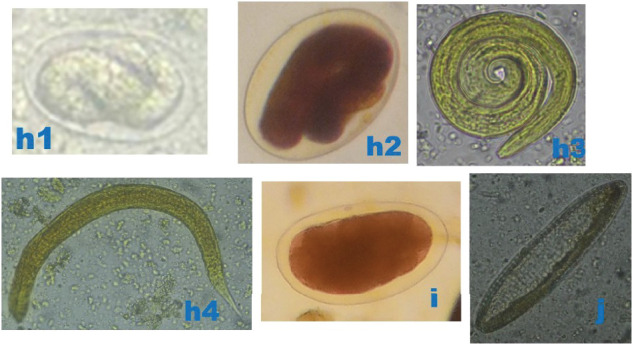
Photomicrographs of helminthic parasites detected in bats. (h1) Thin walled larvated/embryonated ellipsoidal egg of *Strongyloides* sp. (80 × 65 *μ*m), 400×, after floatation technique, in microbat. (h2) Thin walled larvated ellipsoidal egg of *Strongyloides* sp. (86 × 66 *μ*m), 400×, after floatation technique, in megabat. (h3, h4) Coiled and stretched larvae of *Strongyloides* sp., 400×, after floatation technique, in microbat and megabat, respectively. (i) Unidentified Nematode 1 egg (68 × 45 *μ*m), 400×, after floatation technique, in microbat. (j) Unidentified Nematode 2 egg (78 × 30 *μ*m), 400×, after sedimentation technique, in microbat. *Original micrographs by James Robert Ochieng.*

**Figure 6:**
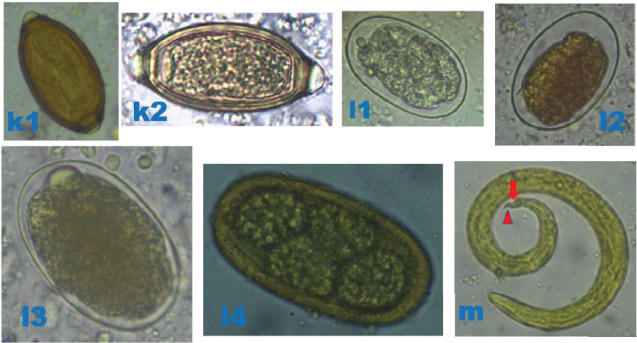
Photomicrographs of helminthic parasites detected in bats. (k1) Brown barrel-shaped *Trichuris* sp. egg (50 × 25 *μ*m), 400×, after sedimentation technique, in microbat. (k2) Barrel-shaped *Trichuris* sp. egg (54 × 30 *μ*m), 400×, after floatation technique, in megabat. Both (k1) and (k2) possess a thick shell and a pair of polar plugs (bipolar protuberances) at each end. (l1, l2) Thin smooth oval-shelled hookworm eggs (55 × 35 *μ*m), 400×, after floatation technique, in microbats and megabats, respectively. (l3) Oval thin smooth-shelled hookworm egg (75 × 45 *μ*m), 400×, after floatation technique, in megabats. (l4) Oval hookworm egg (77 × 40 *μ*m) with four grouped cells ‘morula', 400×, after sedimentation technique, in megabats. (m) *Angiostrongylus* sp. larvae with a dorsal spine and notch (red arrow) and tail terminates in wave-shaped kink (red arrowhead), 400×, after sedimentation technique, in microbats. *Original micrographs by James Robert Ochieng.*

Bat faecal samples (*n* = 484): 242 from Burondo subcounty and 242 from Ntandi town council (Table 1) were tested for parasite eggs, cysts and/or oocytes using standard floatation and sedimentation diagnostic techniques. Thirteen parasite species (Table 1), including protozoa (*n* = 3) (Figure 3), trematode (*n* = 1) (Figure 4), cestode (*n* = 1) (Figure 4) and nematodes (*n* = 8) (Figures 4, 5 and 6), were detected. Of the 484 bat faecal samples examined, 224 (46%) tested positive for more than one parasite species. Amongst the detected parasite species, seven were shared across the four bat groups (Table 1). The most prevalent parasites were *Entamoeba coli* (57%), hookworm (33%), *Strongyloides* sp. (33%) and *Entamoeba histolytica* (32%), and the least prevalent were the two unidentified nematodes (1%) (Table 1).

Whilst the parasite species diversity ranged between 1.67 ≤ H⁣′ ≤ 2.20, parasite species richness ranged from 53.8% to 100%, and the parasite species evenness was 0.65 ≤ J⁣′ ≤ 0.86 (Table 2). The parasite species diversity and evenness lie within the normal standard range of 1.5 ≤ H⁣′ ≤ 3.5 and 0 ≤ J⁣′ ≤ 1, respectively, indicating uniformity in the four studied bat groups [34, 35].

Statistically, a significant difference was detected in the overall GIP prevalence across the four studied bat groups (*X*^2^ [3, *N* = 484] = 213.2, *p* < 0.05) and in the overall prevalence rate of the main potentially zoonotic GIPs of public health importance between the micro- and megabats (*X*^2^ [1, *N* = 484] = 227.4, *p* < 0.05). There was a significant statistical difference (*p* < 0.05) in the prevalence rate of the eight (89%) out of the nine main GIPs of potentially zoonotic public health importance (Table 3). Also, a Mann–Whitney *U* test revealed that seasonality did not have an effect on the parasite prevalence (*p* > 0.05) and that parasite prevalence was significantly higher in microbats compared to megabats (*p* < 0.05) and in Burondo subcounty compared to Ntandi town council (*p* = 0.002).

During the study, the highly detected five microbats included the *Epomophorus* species, *Hipposideros* sp., *Nycteris hispida*, *Pipistrellus nanus* and *Micropteropus pusillus* and four megabats, straw-coloured fruit bats; East African epauletted fruit bats, which were mostly detected in rocky caves; Ethiopian epauletted fruit bats and the Egyptian fruit bats, *Rousettus aegyptiacus*, mostly present in caves. However, because of the highly recognized coexistence/habitat sharing of most microbat species, as was the case amongst megabats, it was hard to differentiate the guano samples per species, but we managed to categorize them into two as microbats and megabats.

## 4. Discussion

From the parasitological point of view, bats are of special interest for several reasons. Bats are susceptible to many infections of animals and humans; they are important vectors and/or reservoirs of pathogens of public health importance [6, 8, 28]. This study determined the diversity and prevalence of bat GIPs in human communities contiguous to SNP, Bundibugyo district. To our knowledge, this is the first extensive research to assess bat GIPs in communities contiguous to protected areas of SNP and Uganda. Of the detected parasites, 77% (*n* = 10) are zoonotically important in the aspect of public health [36], but also important concerning bat/wildlife conservation efforts [6, 8]. To date, there is still little information on the diversity and prevalence of GIPs on several mammalian species of wild fauna despite the currently recognized increase in human–wildlife interaction in the study area [13]. The present study findings are critical in understanding the prevalent parasites that affect the bats' health in the study area and the potentially zoonotic agents they harbour [36].

The overall bat GIP prevalence was 46%. This is low compared to 63.6%, 76%, 76.78%, 80% and 96.29% detected in Arkansas and North Carolina [26], England [37], Nigeria [21], Nepal [10] and Brazil [3], respectively. However, the detected bat GIP prevalence in the present study is higher compared to other previous related findings; namely, [20] reported 43.9% GIPs in bats in Egypt, [38] reported 14% GIPs in bats in Costa Rica and [39] reported zero (0%) prevalence in bats in Ghana.

The recognized differences in GIP prevalence in the current study from previous ones could mostly be attributed to a combination of factors, including but not limited to varying climatic conditions and temperatures, and environmental factors like sanitation that influence the survival, development and spread of parasites [6, 28]. For example, GIPs survive better in the tropical and subtropical regions conducive for the development and transmission of parasitic larvae than in the temperate climates [4]. Other factors may include GIP species and their spread mechanisms: guano sample size, as a larger sample size may have higher chances of more GIP diversity and prevalence, and bat types and their feeding characters, as insectivorous bats encounter and eat many GIP reservoirs, unlike the megabats [8, 28].

Studies have also shown that bats' GIP diversity and prevalence are correlated with their eating habits [20, 40]. Previous related studies by [20, 40] have shown that insectivorous bats acquire parasites by eating GIP reservoirs, including insects (like cockroaches and dung beetles), amphibians, rodents, snails and raw fish [6, 8]. This is the opposite of megabats/fruit eaters [20]. Furthermore, only a few or no cases of trematodes have been detected in fruit bats, including *Rousettus aegyptiacus*, unlike in insectivorous bats [20, 41]. Indeed, the present finding is in line with these previous related studies, where parasite richness was higher in insectivorous bats (92.3%–100%) than in megabats (53.8%–69.2%). Also, the overall GIP prevalence was higher in insectivorous (65%) than in megabats (28%), and only insectivorous bats tested positive for trematode eggs, as in previous related studies by [20, 41].

In the present study, the highly and moderately prevalent parasites, namely, *E*. *coli*, *E. histolytica*, *Eimeria* sp., hookworms, *Strongyloides* sp., *Ascaris* sp. and *Trichuris* sp., are known to have several reservoir hosts [6, 15, 28]. Some also have horizontal transmission, for example, *E*. *coli*, *E. histolytica*, *Eimeria* sp. and *Trichuris* sp. [15]. This is opposite for the low prevalent GIPs like *Fasciola* sp. and *Chitwoodspirura* sp. with indirect life cycles and fewer intermediate hosts [6, 15].

GIP horizontal transmission in bats occurs through the direct ingestion of contaminated food and/or water [8, 28]. Insectivorous bats acquire *Fasciola* sp. by ingestion of infected snails, the intermediate host, during feeding [4]. In a similar case, insectivorous bats acquire *Chitwoodspirura* sp. through ingesting bat flies (families: Nycteribiidae and Streblidae), the obligate ectoparasites of bats, but also intermediate hosts for *Chitwoodspirura* sp. [4]. The recognized *Hymenolepis* sp. parasites in the insectivorous bats could be attributed to their eating of rodents, known to be both reservoir and definitive hosts, but also eating of arthropods, like beetles, the reservoir host [15, 28]. In addition, *Hymenolepis* sp. also has direct transmission and autoinfection competency [15]. *Angiostrongylus* sp. (family Metastrongylidae) is a parasitic nematode with two species, *Angiostrongylus cantonensis* (rat lungworm) and *Angiostrongylus costaricensis*, causing human infections. The former causes eosinophilic meningitis, and the latter leads to abdominal angiostrongyliasis in humans [42], and could be the same in bats. Insectivorous bats acquire *Angiostrongylus* sp. by eating infected snails or slugs, the intermediate hosts [6, 42], though it is not clear how megabats acquire this parasite, as in the present study.

The two unidentified nematodes were only detected in microbats. The unidentified Nematode 1, marked as micrograph ‘i', appeared in the shape of an *Enterobius* species parasite, a known GIP with a zoonotic potential [4]. Also, the unidentified Nematode 2, marked as micrograph ‘j', appears in the shape of an *Oxyurid* sp. egg, as was the case in insectivorous bats in southcentral Nepal by [10], but more interventions are still needed to classify it satisfactorily. Nonetheless, *Oxyurid* species are known GIPs of rodents, and insectivorous bats can acquire infections by eating infected rodents [4]. During the study, the highly recognized human–bat interactions likely to facilitate potentially zoonotic parasite transmission included human activities like mining in rocky bat caves, where we sampled fruit bat roosts. Other risk factors captured through citizen science included bat (bushmeat) hunting for food, the traditional use of bats as medicine and/or for witchcraft, eating bat-nibbled fruits, poor sanitation and a lack of awareness of bat-borne diseases, amongst others, as highlighted in previous related studies [13, 43]. These factors, in addition to the current increase in human populations, causing human–wildlife conflicts and climate change–related factors, also increase risks of bat–human interaction in the study area, increasing potential risks for zoonosis. The detected parasites in this study can negatively impact bats' health, reproductive success and survival, potentially leading to malnutrition and increased disease susceptibility and death [44–46]. The detected bat-borne potentially zoonotic parasites pose a risk to human health [7, 15, 47]. This highlights the importance of understanding the interactions between bats, parasites and humans and the need for community awareness of the bat-mediated health-associated risks in the study area to break the parasite transmission cycle and save human lives from potentially bat-borne zoonotic parasites.

## 5. Limitations and Recommendations

The first limitation relates to matching the detected parasites to specific bat species. This is because of the highly recognized coexistence of most microbat species, as was the case amongst megabats, though we managed to categorize them as micro- and megabats. Secondly, we did not use molecular techniques during parasite diagnosis, so we could not classify most of the parasites to the species level.

Based on the currently increasing changes in habitat, forest fragmentation and urbanization, screening wildlife populations for zoonotic parasites is crucial for public health safety. Nevertheless, studying parasites associated with wildlife offers a significantly different scenario to that of humans and/or domestic animals and contributes to wildlife conservation.

Due to the current findings, future studies could benefit from putting significant effort in classifying the bats and GIPs to species level using molecular techniques. This will shed more light on differentiating the bats and the GIP species. Such data is crucial in public health and biodiversity conservation in Uganda and beyond.

## 6. Conclusions

This study confirms the circulation of potential bat-mediated zoonotic parasites in human communities contiguous to SNP, which pose public health risks. This study, being the first of its kind in Uganda, addresses the knowledge gap in bat GIPs. Therefore, this data highlighted the critical role of bats as sentinel species for effective GIP surveillance in bat hotspot areas. This enhances the need for early detection, control and prevention of parasite zoonosis in human communities, especially in contiguity and/or proximity to the protected areas known to possess many reservoir hosts.

## Figures and Tables

**Table 1:** Prevalence (%) of gastrointestinal parasites diagnosed in bat guano in Burondo subcounty and Ntandi town council, Bundibugyo district, western Uganda.

**Parasite**	**Burondo subcounty**		**Ntandi town council**		**Overall prevalence (%)**	**Chi-square test**	
	**Microbats (** **n** = 121**) n (%)**	**Megabats (** **n** = 121**) ** **n (%)**	**Microbats (** **n** = 121**) n (%)**	**Megabats (** **n** = 121**) n (%)**		**X** ^2^	**p** ** value**
Protozoa							
*Entamoeba coli*	81 (67.8)	67 (55.4)	72 (59.5)	55 (45.5)	57	11.88	0.008
*E*. *histolytica*	37 (30.6)	43 (35.5)	50 (41.3)	26 (21.5)	32	11.43	0.01
*Eimeria* sp.	19 (15.7)	13 (10.7)	20 (16.5)	11 (9.1)	13	4.29	0.232
Trematoda							
*Fasciola* sp.	10 (8.3)	0 (0.0)	3 (2.5)	0 (0.0)	3	21.11	⁣^∗∗∗^
Cestoda							
*Hymenolepis* sp.	21 (17.4)	0 (0.0)	5 (4.1)	0 (0.0)	5	48.29	⁣^∗∗∗^
Nematoda							
Hookworm	59 (48.8)	19 (15.7)	72 (59.5)	9 (7.4)	33	104.41	⁣^∗∗∗^
*Trichuris* sp.	37 (30.6)	9 (7.4)	33 (27.3)	11 (9.1)	19	34.67	⁣^∗∗∗^
*Ascaris* sp.	43 (35.5)	14 (11.6)	36 (29.8)	5 (4.1)	20	49.39	⁣^∗∗∗^
*Strongyloides* sp.	68 (56.2)	22 (18.2)	53 (43.8)	19 (15.7)	33	63.72	⁣^∗∗∗^
*Angiostrongylus* sp.	11 (9.1)	2 (1.7)	7 (5.8)	0 (0.0)	4	15.44	0.001
*Chitwoodspirura* sp.	10 (8.3)	1 (0.9)	2 (1.7)	0 (0.0)	3	19.84	⁣^∗∗∗^
Unidentified Nematode 1	2 (1.7)	0 (0.0)	1 (0.9)	0 (0.0)	1	3.69	0.297
Unidentified Nematode 2	0 (0.0)	0 (0.0)	4 (3.3)	0 (0.0)	1	12.1	0.007

*Note: n* = number of bat faecal samples examined, % = percentage prevalence, *X*^2^ = chi-square value, *p* value = chi-square *p* value.

Abbreviation: sp., species.

⁣^∗∗∗^*p* value less than 0.001.

**Table 2:** Bat gastrointestinal parasite richness, diversity and evenness in micro- and megabats in Burondo subcounty and Ntandi town council, Bundibugyo district, western Uganda.

**Bat type and study area**	**Detected parasites (** **n** **)**	**Parasite richness**	**Diversity index (H**⁣′**)**	**Evenness (J**⁣′**)**
Microbats Burondo	12	12/13 (92.3%)	2.20	0.86
Megabats Burondo	9	9/13 (69.2%)	1.78	0.69
Microbats Ntandi	13	13/13 (100%)	2.09	0.81
Megabats Ntandi	7	7/13 (53.8%)	1.67	0.65

**Table 3:** Prevalence rate (%) of the main potentially zoonotic GIPs of public health importance detected in the micro- and megabat guano in Burondo subcounty and Ntandi town council, Bundibugyo district, western Uganda.

**Parasite**	**Microbats (** **n** = 242**)**	**Megabats (** **n** = 242**)**	**Chi-square test**	
	**n** ** (%)**	**n** ** (%)**	**X** ^2^	**p** ** value**
*Entamoeba histolytica*	87 (36)	69 (29)	3.07	0.080
*Fasciola* sp.	13 (5)	0 (0)	13.36	⁣^∗∗∗^
*Hymenolepis* sp.	26 (11)	0 (0)	27.48	⁣^∗∗∗^
Hookworm	131 (54)	28 (12)	99.37	⁣^∗∗∗^
*Trichuris* sp.	70 (29)	20 (8)	34.12	⁣^∗∗∗^
*Ascaris* sp.	79 (33)	19 (8)	46.06	⁣^∗∗∗^
*Strongyloides* sp.	121 (50)	41 (17)	59.38	⁣^∗∗∗^
*Angiostrongylus* sp.	18 (7)	2 (1)	13.35	⁣^∗∗∗^
*Chitwoodspirura* sp.	12 (5)	1 (0)	9.57	0.002

*Note: n* = number of bat faecal samples examined, % = percentage prevalence, *X*^2^ = chi-square value, *p* value = chi-square *p* value.

Abbreviation: sp., species.

⁣^∗∗∗^*p* value less than 0.001.

## Data Availability

The data supporting this study's findings are available from the corresponding author upon request.
